# Optimization of the cydex blue assay: A one-step colorimetric protein assay using cyclodextrins and compatible with detergents and reducers

**DOI:** 10.1371/journal.pone.0195755

**Published:** 2018-04-11

**Authors:** Thierry Rabilloud

**Affiliations:** Chemistry and Biology of Metals, UMR5249, Univ. Grenoble Alpes, CNRS, CEA, BIG-LCBM, Grenoble, France; INRA, FRANCE

## Abstract

Sodium dodecyl sulfate electrophoresis (SDS) is a protein separation technique widely used, for example, prior to immunoblotting. Samples are usually prepared in a buffer containing both high concentrations of reducers and high concentrations of SDS. This conjunction renders the samples incompatible with common protein assays. By chelating the SDS, cyclodextrins make the use of simple, dye-based colorimetric assays possible. In this paper, we describe the optimization of the assay, focussing on the cyclodextrin/SDS ratio and the use of commercial assay reagents. The adaptation of the assay to a microplate format and using other detergent-containing conventional extraction buffers is also described.

## Introduction

Determination of protein concentration is one of most common assays used in biochemistry. The most commonly used protein assays belong to two families. In the first family (copper based methods), the biuret reaction is used to detect the presence of peptide bonds. In this reaction, Cu(II) is reduced to Cu(I) by peptide bonds in strongly alkaline conditions. The resulting Cu(I), the amount being proportional to the amount of peptide bonds, is then measured either by reduction of Folin-Ciocalteu’s reagent [1], or by the formation of a colored complex with bicinchoninic acid [2].

The second family of methods (dye-binding methods) is based on the ability of proteins to bind to certain dyes and to induce an absorption shift in the dyes. The most common method uses Coomassie blue as the dye [3], but metal-dye complexes, such as pyrocatechol-molybdate [4] or pyrogallol red-molybdate [5] have also been used for protein assays.

The chemical bases of these two methods, in turn, determine the chemicals that will interfere with the protein assay.

On the one hand, as the copper-based methods rely on the reduction of Cu(II) to Cu(I), reducers present in the sample will induce a strong interference by reducing copper, even in the absence of any protein. Such reducers include those naturally present in the sample (e.g. ascorbate), but also reducers intentionally included in the extraction buffer to maximize protein extraction, for example disulfide bond reducers, such as mercaptoethanol or dithiothreitol.

On the other hand, proteins are not the only chemicals able to induce an absorption shift in dyes. By changing the characteristics of the milieu, most detergents used in biochemistry also induce a dye absorption shift and thus interfere strongly with the dye-binding methods. In addition, when present in high concentrations, some detergents interfere with the biuret reaction and, thus, with copper-based methods.

As two very different protein assays are available, it is often possible to select one method that is not sensitive to interference from the sample or the sample extraction process. However, there are some exceptions to this rule, which raise concerns. One example is the classical Laemmli type SDS buffer, which contains high concentrations of SDS (generally 2% w/v) and high concentrations of reducers (usually 5% mercaptoethanol or 50 mM dithiothreitol). While this conjunction yields excellent solubilizing power for almost all proteins, it renders the extracts intractable to simple protein assays.

Another buffer that presents difficulties is the Radio-Immuno Precipitation Assay (RIPA) buffer, which contains a mixture of ionic and non-ionic detergents. A classical RIPA buffer formulation contains 1% w/v of NP40/Igepal (non-ionic detergent), and a mixture of 0.5% sodium deoxycholate and 0.1% SDS (ionic detergents). Compared to the SDS buffers, this buffer has a lower, albeit important, solubilizing power. It does not solubilize cell nuclei, thereby avoiding DNA release and thus the associated marked increase in viscosity. The RIPA buffer is one example of a buffer that interferes in both types of protein assays by means of the detergent only.

These two extraction buffers are very popular prior to SDS electrophoresis, which is often coupled to specific protein detection methods, such as western blot techniques. Comparison of western blot signals implies to be able to normalize the signals [6], and it has been demonstrated that the best normalization is achieved through general detection of the proteins loaded in the SDS electrophoresis lanes [7–11].

However, because of the saturation and detection threshold phenomena, even this normalization method requires that comparable protein amounts are loaded in the gel lanes for analysis, which implies in turn to be able to determine the protein concentrations in samples dissolved in SDS or in RIPA buffer.

A solution to this problem is to precipitate the proteins in the sample so as to remove the interfering substances, and then to redissolve the protein pellet in an assay-compatible buffer and to perform the assay [12–14]. However, such methods are more cumbersome than direct protein assays and also more prone to errors induced by variability in the precipitation step.

Recently, a direct, single-step method of measuring protein concentrations has been described [15]. This method is based on the ability of cyclodextrins to form complexes with hydrophobic [16] and amphiphilic molecules, such as detergents [17–19]. As this method involves the use of large volumes of cyclodextrin solutions in order to accommodate large volumes of SDS-containing buffers, it requires the use of a dye-reagent concentrate which is both corrosive and viscous and, therefore, not easy to use. Moreover, this method could not be used in the microplate format, which has become popular for protein assays. A modification of this method, compatible with normal strength Bradford protein reagents and compatible with the microplate format, is described in this manuscript.

## Material and methods

All experiments described in this paper were performed as independent duplicates.

### Reagents

Alpha-cyclodextrin (e.g. Sigma, catalog number #28705) was dissolved at 125mg/ml in ultrapure water. Beta-cyclodextrin (e.g. Alfa, catalog number # A14529) was dissolved at 15mg/ml in ultrapure water. Dissolution was facilitated by warming the water prior to adding it to the cyclodextrin powder.

#### Bradford reagent

A commercial concentrate (Bio Rad, catalog number #500–0006) was used. This reagent was diluted 5-fold in water to prepare a working reagent comparable to other commercial Bradford reagents. For some experiments, a semi-concentrated (2 x strength) reagent was prepared by adding 20 ml of the commercial concentrate to 30 ml of ultrapure water.

#### Protein standards

Bovine serum albumin (Sigma, catalog number #A6003), hen conalbumin (Sigma, catalog number #C0755), soybean trypsin inhibitor (Sigma, catalog number #93620) and hen lysozyme (Sigma, catalog number #62971) were each dissolved at 10 mg/ml in ultrapure water. To mimic the situation prevailing in SDS electrophoresis, the standards were further diluted at the adequate protein concentration in SDS sample buffer (2% (w/v) SDS, 5% (v/v) beta mercaptoethanol, 10% (v/v) glycerol and 62.5 mM Tris-HCl buffer pH 6.8) using a 2x concentrated stock buffer. To mimic the situation prevailing in RIPA extraction, the standards were further diluted at the adequate protein concentration in RIPA buffer (1% (v/v) IGEPAL 630, 0.5% sodium deoxycholate, 0.1% SDS, 150mM sodium chloride and 50 mM Tris-HCl buffer, pH 8.0) using a 2x concentrated stock buffer. Serial dilutions were used so that the various amounts of standards needed to build the dose-response curves were obtained at fixed sample volumes.

### Methods

#### Protein assay in cuvette format

A complete protein assay reagent was prepared by mixing the required volume of cyclodextrins with commercial, normal strength Bradford reagent (usually 10–50μl of concentrated cyclodextrin solution per ml of Bradford reagent, see Results section).

For each point of the assay, 1 ml of this reagent was pipetted in a 1.5 ml or 2 ml polypropylene centrifuge tube. Aliquots of the sample (5 or 10 μl in water, SDS buffer or RIPA buffer) were then added and the contents mixed by several inversions of the tube. The color was let to develop for at least 5 minutes at room temperature, and the absorbance was read at 595 nm. A zero tube (using buffer only) was performed, and the spectrophotometer blank was obtained with ultrapure water.

#### Protein assay in microplate format

Flat bottom, polystyrene 96-well plates were used. The complete protein assay reagent contained a higher amount of cyclodextrins (up to approximately 90 μl of the concentrated cyclodextrin solution per ml of Bradford reagent; see Results section). 250 μl of this reagent was pipetted into each well, and 5 or 10 μl of the protein sample (in water, SDS buffer or RIPA buffer) was then added. The contents of each well were mixed by repeated pipetting. The color was left to develop for at least 5 minutes at room temperature, and the absorbance was then read at 595 nm. A zero tube using buffer only was performed, and the plate reader blank was obtained against air.

#### Complex samples

RAW264.7 cells were grown in RPMI1640 medium supplemented with 10% fetal bovine serum. The cells were harvested by scraping them from the culture medium, then collected by centrifugation at 100 *g* for 5 minutes, and rinsed twice with PBS. The volume of the cell pellet was then estimated, and the protein extracted by ten equivalent volumes of extraction solution. Different extraction solutions were tested as follows:

i) urea extraction solution: 7 M urea, 2 M thiourea, 4% CHAPS, 30 mM Spermine base, 60 mM HCl and 5 mM tris-carboxyethyl phosphine (TCEP). After extraction at room temperature for 1 hour, the extract was centrifuged at 15000 *g* for 15 minutes at 20 °C. The supernatant was then collected.
ii) native extraction solution: 10 mM Hepes-NaOH (pH 7.5), 2 mM MgCl_2_, 50 mM KCl and 0.1% 3-[tetradecyl dimethylammonio]-1-propanesulfonate (SB 3–14). After extraction for 30 minutes on ice, the extract was centrifuged at 15,000 *g* for 15 minutes at 4 °C. The supernatant was then collected.
iii) RIPA buffer: the cell pellet was extracted with RIPA buffer (see above) on ice for 30 minutes. The extract was centrifuged at 15,000 *g* for 15 minutes at 4 °C. The supernatant was then collected.
iv) SDS buffer: samples were extracted with SDS buffer (see above), denatured at 70 °C for 30 minutes and then centrifuged at 15,000 *g* for 15 minutes at room temperature. The viscous supernatant was collected and sheared by repeated passage through a 0.8-mm diameter syringe needle.

Protein assay: the protein concentration of the extracts was determined using the Bradford assay after appropriate dilution (5- or 10-fold dilution) in the corresponding extraction buffer to fit the linear part of the standard.

Urea and native extracts: 5 μl of diluted extract in 1 ml of Bradford reagent.

SDS extracts: 10 μl of diluted extract in 1 ml of Bradford reagent containing alpha-cyclodextrin at 2.5 mg/ml.

RIPA extracts: 10 μl of diluted extract in 1 ml Bradford reagent containing alpha-cyclodextrin at 5 mg/ml.

SDS electrophoresis: The samples obtained from RAW 264 cells were diluted at 1 mg/ml (as determined by the protein assays) in SDS sample buffer to which bromophenol blue had been previously added (0.004% w/v). Samples extracted under conditions (ii) and (iii) were denatured at 70 °C for 30 minutes prior to use; this step was omitted for samples extracted under conditions (i) and (iv). Protein samples (20 or 40 μg) were loaded on top of a large 10% acrylamide gel (160×200×1.5 mm), operated in the tris-taurine system [20]. The empty wells separating the protein-loaded wells were loaded with an equal volume (40 μl) of SDS sample buffer. After electrophoresis at 12 W/gel and 10°C, the gels were stained with colloidal Coomassie blue [21] for 24 hours. The resulting image was scanned using a flatbed scanner (Epson perfection V750) with 48-bit color image acquisition. The staining intensity for each lane was determined using ImageJ software (version 10.2).

## Results

### Classical detergent-containing buffers alter the Bradford protein assay in different ways

**Fig 1:**
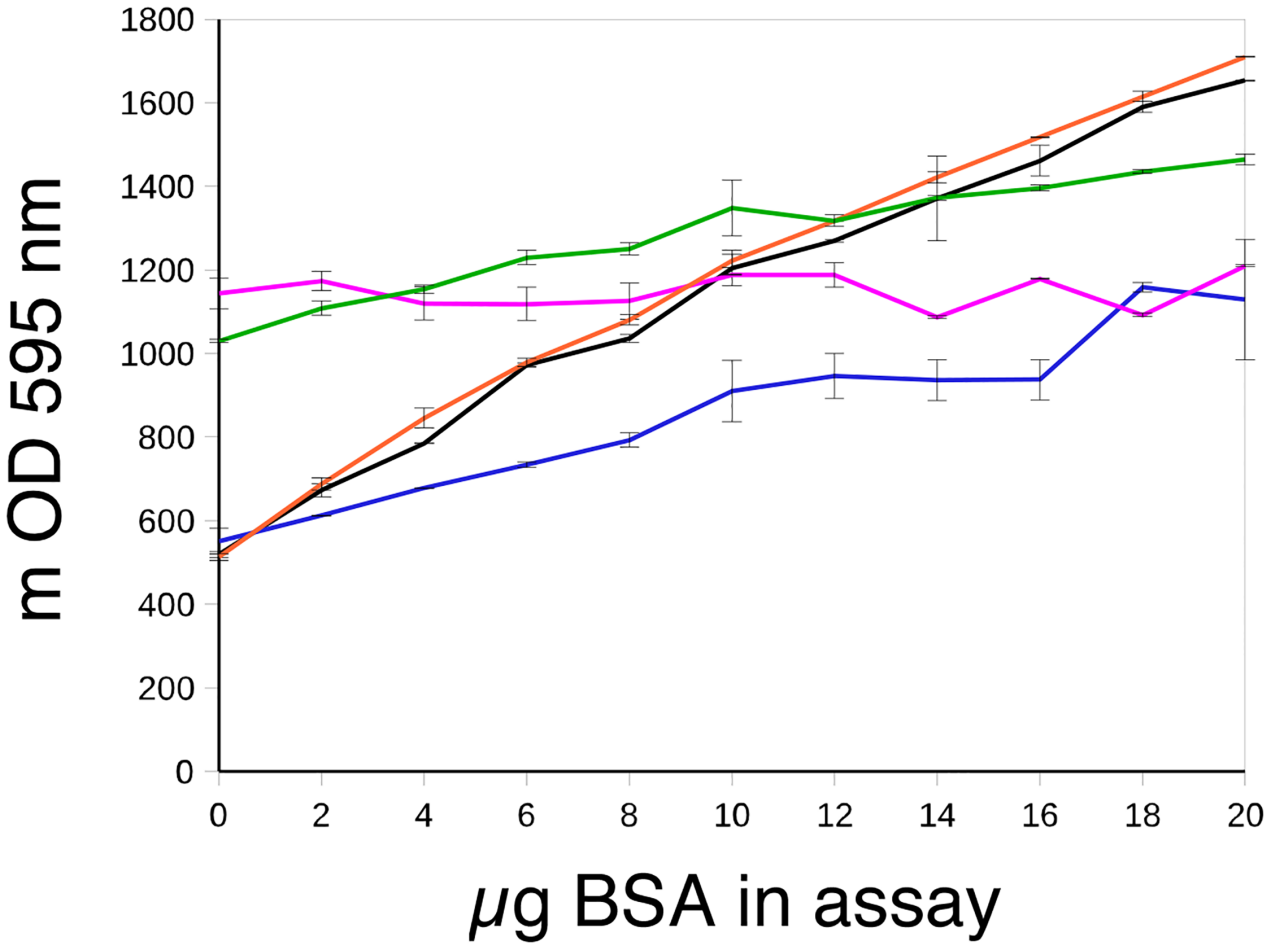
Interference of detergent-containing buffers with the Bradford protein assay. Different concentrations of BSA were assayed with the Bradford reagent under different conditions: Black: control conditions (BSA dissolved in water). Blue: assay with 1 μl of Laemmli-type SDS buffer (2% SDS and 5% mercaptoethanol)/ml of assay mixture. Purple: assay with 10 μl of Laemmli-type SDS buffer (2% SDS and 5% mercaptoethanol)/ml of assay mixture. Orange: assay with 1 μl of RIPA buffer (1% Igepal, 0.5% deoxycholate and 0.1% SDS)/ml of assay mixture. Green: assay with 10 μl of RIPA buffer (1% Igepal, 0.5% deoxycholate and 0.1% SDS)/ml of assay mixture.

The interference of detergents with the Bradford paper has been described since the assay was first published [3]. However, this effect has been investigated for high (in the 0.1–1% w/v range) [3] or very low (in the microgram/ml range) [22] concentrations of detergents. When concentrated protein extracts are available, which is often the case for studies using western blot techniques, the sensitivity of the protein assay allows the use of minute amounts of detergent-containing samples, which do not induce high background interference. To investigate this aspect, the interference of Laemmli-type SDS sample buffer and RIPA buffer was investigated using these buffers at 1 and 10 μl/ml in the final assay in the cuvette format. The results, displayed in Fig 1, show that 10 μl of these detergent-containing buffers produced a high background and a flat response curve, making the assay impossible to conduct. However, the situation was subtler for the lower (1 μl) amount. While the RIPA buffer gave a response curve very similar to the control one, the SDS buffer gave a much flatter response curve, indicative of a low-performance assay as a result. Because of these two phenomena, namely a flat response curve and/or high background, a direct protein assay of samples dissolved in Laemmli-type SDS sample buffer using the Bradford method is not considered to be practical.

### Cyclodextrins decrease SDS interference in the Bradford assay

**Fig 2:**
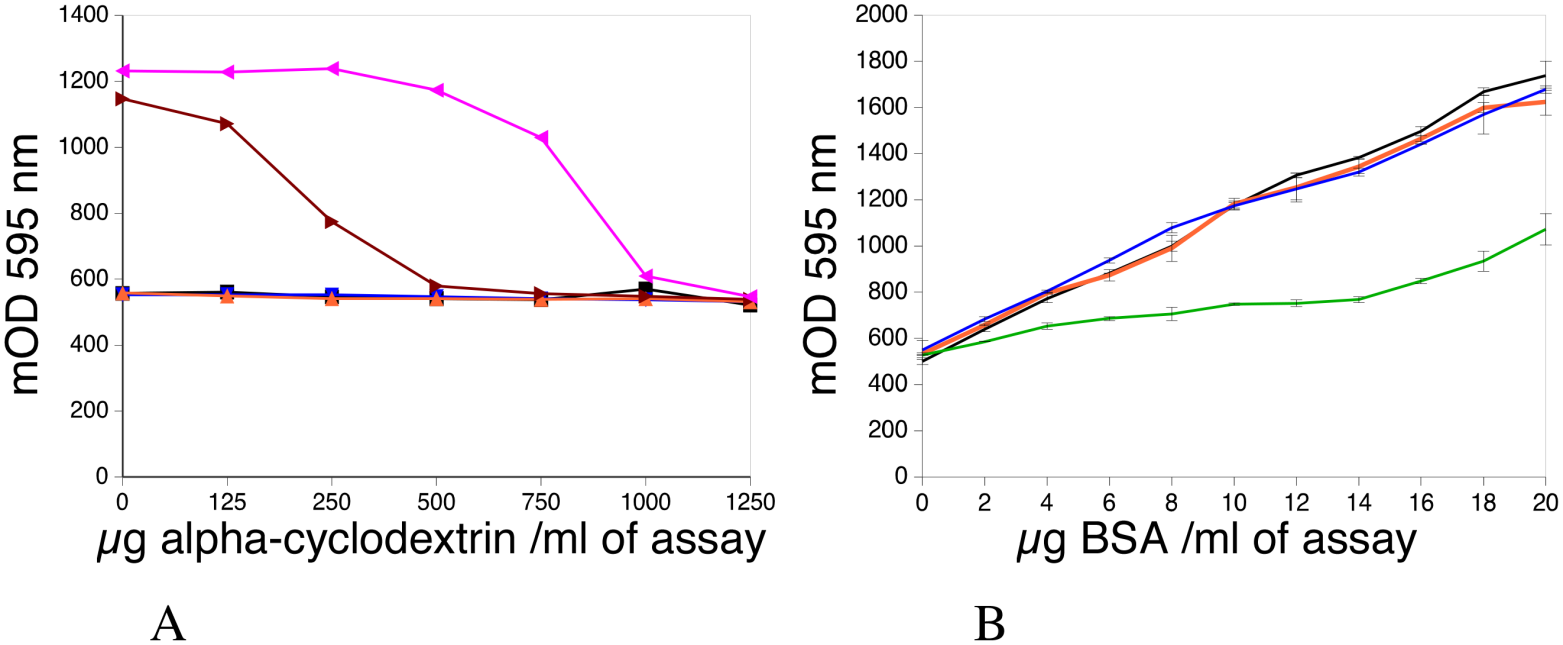
Effect of alpha-cyclodextrin on the Bradford assay. Panel A: effect of cyclodextrin on the background induced by SDS in the Bradford assay. Different concentrations of Laemmli-type SDS buffer were added to the Bradford reagent, together with different amounts of alpha-cyclodextrin. Black: control (no added detergent). Blue: 1 μl of SDS buffer per ml of assay mixture. Orange: 2 μl of SDS buffer per ml of assay mixture. Brown: 5 μl of SDS buffer per ml of assay mixture. Purple: 10 μl of SDS buffer per ml of assay mixture. Panel B: effect of cyclodextrin on the Bradford assay in the cuvette format (1 ml of assay). BSA was assayed by the Bradford assay in the presence of different amounts of cyclodextrin and of SDS-containing buffer. Black: control (no SDS, no cyclodextrin). Orange: 5 μl of SDS buffer, 1.25 mg/ml alpha cyclodextrin in Bradford reagent. (CD/SDS molar ratio: 3.65). Green: 10 μl of SDS buffer, 1.25 mg/ml alpha cyclodextrin in Bradford reagent. (CD/SDS molar ratio: 1.82). Blue: 10 μl of SDS buffer, 2.5 mg/ml alpha cyclodextrin in Bradford reagent (CD/SDS molar ratio: 3.65).

Cyclodextrins are known to form quasi-equimolecular complexes with detergents [17, 19]. It was therefore investigated if formation of this complex could decrease the SDS-induced background in the Bradford assay. The results, displayed in Fig 2A, showed that the addition of alpha-cyclodextrin progressively decreased the SDS-induced background. The background reached control levels at an alpha-cyclodextrin/SDS mass ratio of 5, i.e. a molar ratio of 1.46, which is close to the 1.2 value reported in the literature [17, 19]. However, experiments showed that simple background ablation was not sufficient to restore the assay performance, as shown in Fig 2B and Table 1. At a low alpha-cyclodextrin/SDS molar ratio (<2), the response curve was much flatter than in a standard assay. However, a normal response curve was restored at higher alpha-cyclodextrin/SDS molar ratios (≥3).

**Table 1:** Effect of the cyclodextrin/SDS ratio on the assay response.

**α**-cyclodextrin/SDS	0	1.85	2	2.5	3	3.5	3.7
molar ratio							
blank value (mean)	497.5	527.5	505.5	519	508.5	524	532.5
std dev blank	0.71	0.70	3.53	5.66	0.70	5.65	4.95
value for 10μg BSA		860	887	1087.5	1161.5	1223	1208
std dev 10μg BSA		42.42	31.11	27.58	0.71	9.90	45.25

Values are given in mOD 595 nm. All experiments performed in duplicate

### Alpha- and beta-cyclodextrin do not have the same efficiency on SDS interference

The different cyclodextrins have different physico-chemical characteristics, as reviewed in [23]. In particular, the size of the internal cavity increases from alpha- to beta- (and then gamma-) cyclodextrins. This means that beta-cyclodextrin should bind detergents more effectively than alpha-cyclodextrin, especially bulky detergents such as those used in the RIPA buffer. However, the Coomassie blue molecule used in the Bradford assay is also partly hydrophobic and may be bound by beta-cyclodextrin. Consequently, this binding may induce the color shift used in the assay. The ability of alpha- and beta-cyclodextrin to induce background in the Bradford assay was investigated.

**Fig 3:**
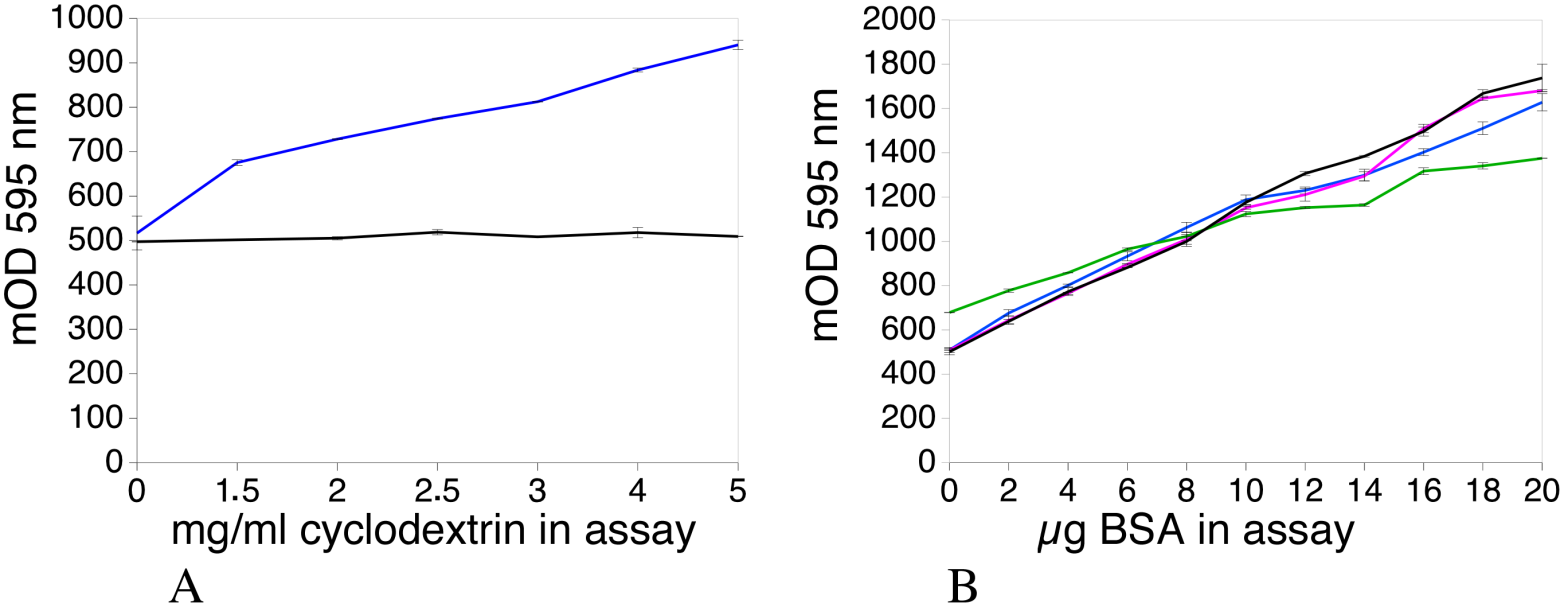
Comparison of the effects of alpha and beta cyclodextrin. Panel A: effect of increasing concentrations of cyclodextrin on the background absorbance of the assay. Different concentrations of alpha-cyclodextrin (black curve) and beta-cyclodextrin (blue curve) were added to the Bradford reagent and the interference measured. Panel B: effects of cyclodextrins in the Bradford assay. Different concentrations of cyclodextrins were used in a protein assay containing 10 μl SDS buffer per ml of assay. Black: control: no SDS buffer, no cyclodextrin. Blue: 2.5 mg alpha-cyclodextrin / ml of Bradford reagent (CD/SDS molar ratio: 3.65). Purple: 5 mg alpha-cyclodextrin / ml of Bradford reagent (CD/SDS molar ratio: 7.3). Green: 2.5 mg beta-cyclodextrin / ml of Bradford reagent (CD/SDS molar ratio: 3.17).

The results, displayed in Fig 3A, showed that increasing amounts of beta-cyclodextrin induce a progressive increase in the background absorbance in the assay, while alpha-cyclodextrin is basically without effect. Furthermore, as shown in Fig 3B, beta-cyclodextrin produces a flatter response curve compared to the control assay or the assay using alpha-cyclodextrin; this results in lower performances.

### The combined use of cyclodextrins and SDS normalizes the protein response in the Bradford assay

**Fig 4:**
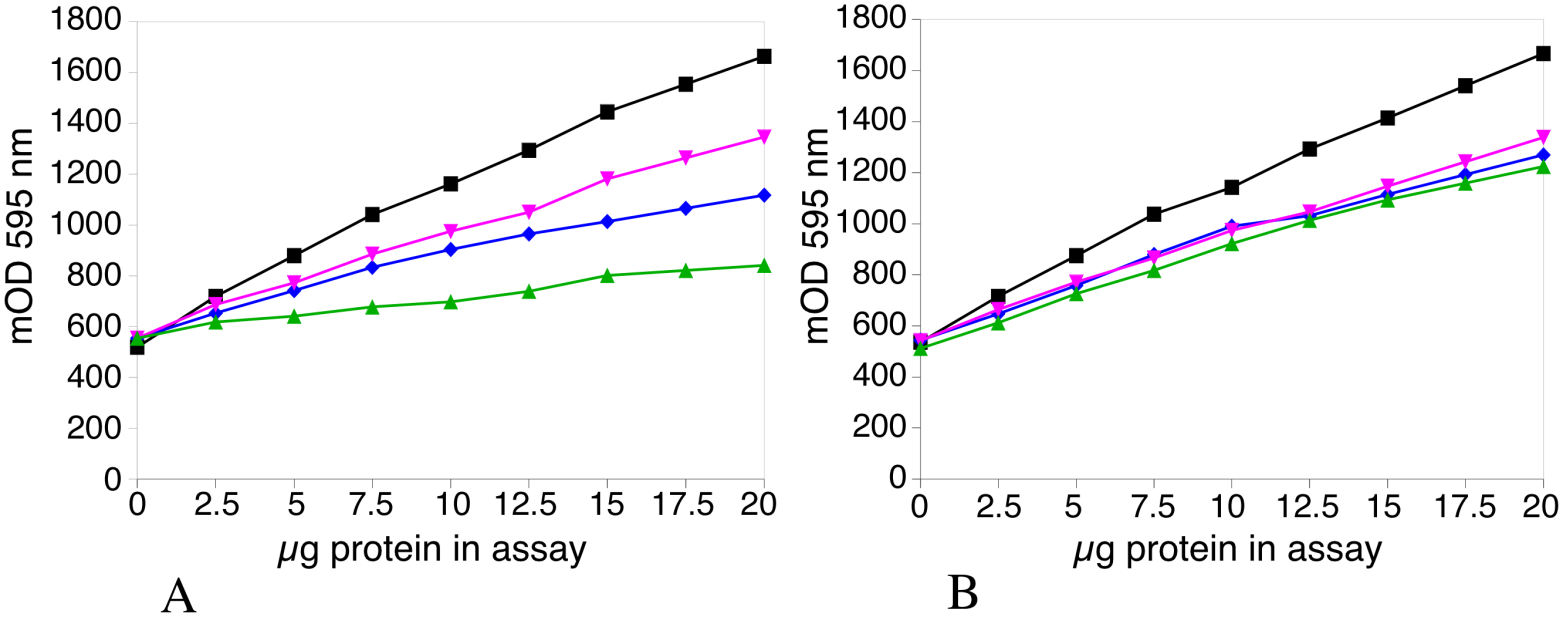
Responses of different proteins in the Bradford assay. Different proteins were assayed by the Bradford method using the cuvette format (1 ml of assay) under control conditions (panel A) or after denaturation in 10 μl of SDS buffer and in the presence of 2.5 mg alpha-cyclodextrin/ ml of Bradford reagent (panel B). Black: bovine serum albumin. Purple: hen conalbumin. Blue: soybean trypsin inhibitor. Green: hen lysozyme.

The responses curves for different proteins in the Bradford assay are known to be different [24]. These differences are decreased when denaturing treatments are applied to the proteins, either with a base [25] or with urea [22]. The combined effect of SDS (for protein denaturation) and cyclodextrin (to remove SDS interference) was thus investigated using four different proteins. As shown of Fig 4, the combined use of SDS and cyclodextrin decreases the protein-to-protein variability of the assay, although BSA retained a higher response curve than the other proteins. This means that BSA is probably not the best standard protein to get a response closer to an absolute quantification, as noted in previous papers that suggested the use of ovalbumin [26]. BSA, however, is still a valuable standard for relative quantification.

### Use of cyclodextrins for protein assay in the RIPA buffer

**Fig 5:**
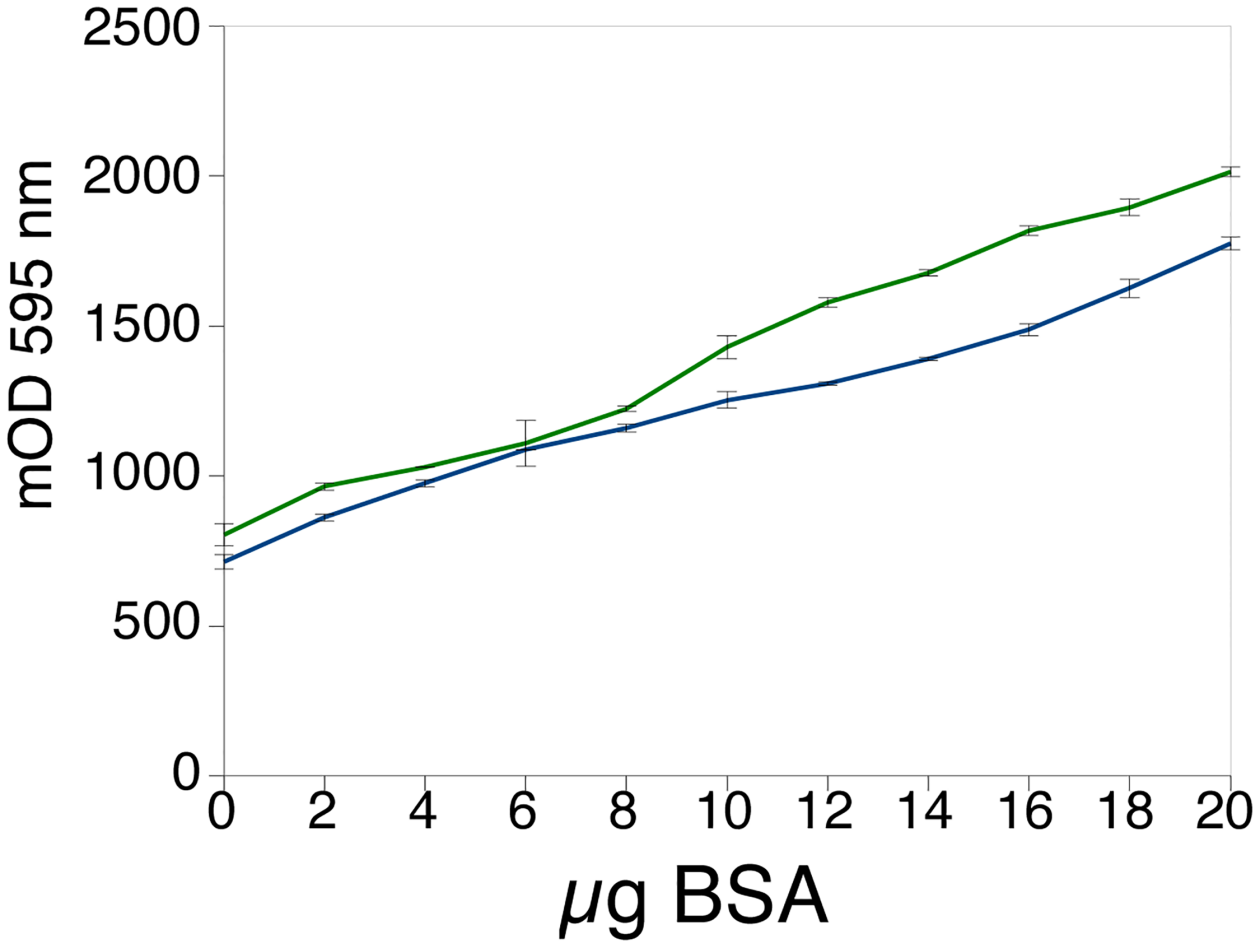
Effect of cyclodextrins on the protein assay in the presence of RIPA buffer. BSA was assayed in 10μl of RIPA buffer in the cuvette format (1ml). Blue: assay in the presence of 5mg/ml alpha cyclodextrin. Green: assay in the presence of 2.5 mg/ml beta cyclodextrin.

The RIPA buffer contains a mix of linear (SDS) and bulky (Igepal/Triton, deoxycholate) detergents, which makes it difficult to use in the Bradford assay. Theoretically, beta-cyclodextrin should be more effective than alpha-cyclodextrin for this buffer, as it is able to bind larger and bulkier molecules. However, as shown in Fig 3, beta-cyclodextrin causes some problems when used in the Bradford assay. As alpha-cyclodextrin is not associated with these problems and is much more water-soluble than beta-cyclodextrin, its use in the protein assay, with the RIPA buffer, was investigated. Preliminary experiments (Table 2) confirmed that low amounts of alpha-cyclodextrin did not remove the detergent-induced background absorbance. However, increasing the alpha-cyclodextrin concentration in the assay to 5 mg/ml or more decreased the background absorbance while retaining an adequate response. This was further verified, as shown in Fig 5, indicating that an assay using alpha-cyclodextrin can be efficiently designed to measure protein concentrations in RIPA buffer.

**Table 2:** Effect of the cyclodextrin concentration in an assay containing RIPA buffer.

**α**-CD (mg/ml)	2.5	5	7.5	10
blank (mean)	945.5	741.5	676	615.5
std dev blank	0.71	10.61	21.21	36.06
value 5 μg BSA	1252	1133.5	1040	1013
std dev 5 μg BSA	24.04	33.23	28.28	5.66
Δ OD 5 μg BSA	306.5	392	364	397.5
value 10 μg BSA	1592	1480.5	1342.5	1289
std dev 10 μg BSA	22.63	17.68	65.76	26.87
Δ OD 10 μg BSA	646.5	739	666.5	673.5
value 20 μg BSA	2084	2079	1850	1795.5
std dev 20 μg BSA	35.35	8.48	15.56	13.43
ΔOD 20 μg BSA	1138.5	1337.5	1174	1180

Values are given in mOD 595 nm. All experiments performed in duplicate

Assay performed in the cuvette format (1ml) with 10 μl RIPA buffer

### Adaptation of the cyclodextrin-Bradford assay to the microplate format

As shown in Figs 2 and 5, the efficiency of the assay is linked to the alpha-cyclodextrin/SDS ratio, which must be above a given, empirical value. This is easy to achieve in the cuvette format, where the sample is diluted 100-fold in the Bradford reagent. However, this may be more difficult to achieve in the microplate format where the sample volume must be kept within the 5–10μl range and the reagent volume is reduced to 250 μl.

Maintaining the cyclodextrin/SDS ratio may be achieved in one of two ways. The first and easiest technique is to dilute the concentrated alpha-cyclodextrin solution into a normal strength Bradford reagent and to use 250 μl of this complete reagent. The second and more precise method is to use a reagent concentrate and to dilute this with water and the concentrated alpha-cyclodextrin solution to obtain a working reagent that will have the required cyclodextrin concentration and still be normal strength.

**Fig 6:**
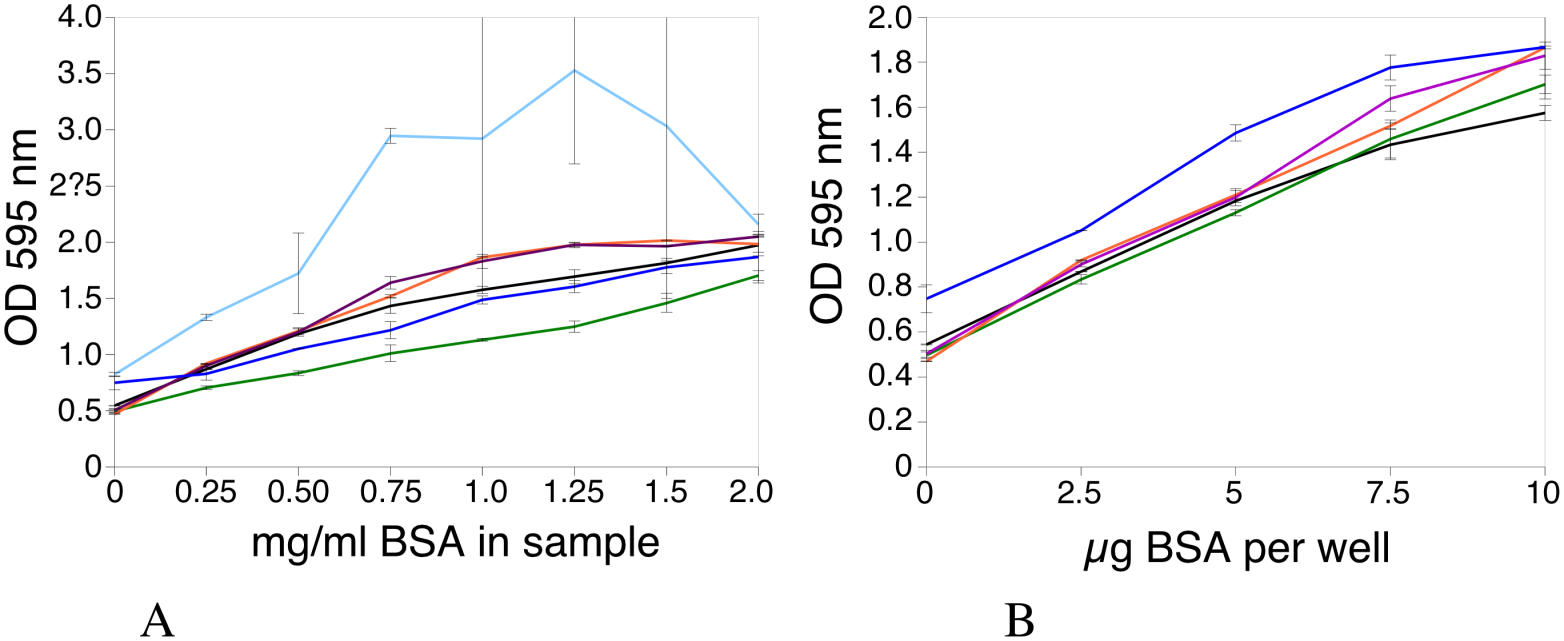
Protein assay in the microplate format (250μl). BSA dilutions were assayed using the microplate format. Black: 10 μl of BSA dilutions in water. Green: 5 μl of BSA dilutions in SDS buffer, reagent made by adding 10 μl alpha-cyclodextrin solution (125 mg/ml) to 240 μl of Bradford reagent. Orange: 10 μl of BSA dilutions in SDS buffer, reagent made by adding 20 μl alpha-cyclodextrin solution (125 mg/ml) to 230 μl of Bradford reagent. Brown: 10 μl of BSA dilutions in SDS buffer, reagent made by adding 20 μl alpha-cyclodextrin solution (125 mg/ml) to 105 μl of water and 125 μl of 2× concentrated Bradford reagent. Blue: 5 μl of BSA dilutions in RIPA buffer, reagent made by adding 20 μl alpha-cyclodextrin solution (125 mg/ml) to 230 μl of Bradford reagent. Light blue: 10 μl of BSA dilutions in RIPA buffer, reagent made by adding 40 μl alpha-cyclodextrin solution (125 mg/ml) to 85 μl of water and 125 μl of 2× concentrated Bradford reagent. This special reagent was tried to normalize the response of the assay to 10μl RIPA buffer. In panel A, the abscissae values are the concentration of BSA (in mg/ml) of the sample, which can be of 5 or 10μl size depending on the assay conditions. In panel B, the abscissae values are the amount of BSA per well.

Both of these methods were investigated and the results are presented in Fig 6. In the case of the Laemmli buffer, the two strategies produced equivalent results, and both sample sizes (5 and 10μl) produced equivalent results when renormalized for protein amount (Fig 6B).

In the case of the RIPA buffer, a 5-μl sample size is the only practicable one, the assay being completely erratic for the 10 μl sample size. This may be due to the very high cyclodextrin concentration (approximately 20 mg/ml) that is required in this assay.

### Practical use for complex samples

For getting a first view of the performances of the assay, a false blind experiment was performed. It consisted in reporting the values obtained for known amounts of BSA on a standard curve obtained from another series of values in a different experiment. The results of this experiment are reported in Table 3. It shows that the modified versions of the Bradford assay are not more variable than the standard assay.

**Table 3:** False blind experiments for assessing the accuracy of the assay.

Regular Bradford											
theoretical value (μg protein)	0	2	4	6	8	10	12	14	16	18	20
observed value (μg protein)	0	1.19	3.05	5.11	6.98	8.69	12.86	14.32	16.85	16.92	18.98
% error	N/A	40.34	23.63	14.76	12.76	13.09	7.15	2.29	5.31	5.97	5.09
Laemmli buffer with **α**-CD											
theoretical value (μg protein)	0	2	4	6	8	10	12	14	16	18	20
observed value (μg protein)	0	2.08	4.05	6.07	8.23	9.81	12.28	14.38	16.69	19.04	20.88
% error	N/A	3.88	1.37	1.18	2.89	1.91	2.36	2.72	4.31	5.79	4.42
RIPA Buffer with **α**-CD											
theoretical value (μg protein)	0	5	10	20							
observed value (μg protein)	0	3.62	8.76	16.99							
% error	N/A	27.64	12.37	15.06							

Note: the values used to calculate the « practical » amounts (here named observed values) were obtained in different experiments than those used to build the standard curve. This means in turn that the experiment–to-experiment variability is included in the error.

**Fig 7:**
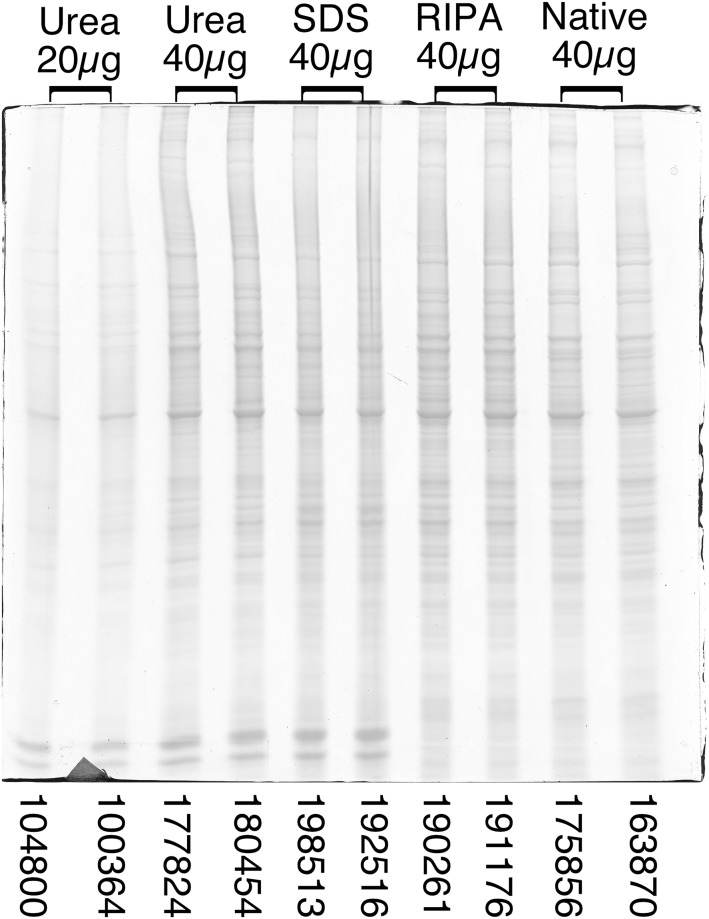
SDS electrophoresis experiment made with various detergent containing extraction solutions. RAW264 cells were extracted under various conditions (details reported in Material and methods). Urea: 7 M urea, 2 M thiourea, 4% CHAPS. SDS: 2% SDS, 5% mercaptoethanol. RIPA: 1% Igepal, 0.5% deoxycholate, 0.1% SDS. Native: 0.1% SB 3–14.

To go beyond standard proteins into complex cell biology samples, SDS electrophoresis and related techniques (e.g. western blotting) are the most likely techniques able to take advantage of the possibility offered by this assay to measure protein concentrations in Laemmli-type SDS sample buffer and in RIPA buffer, and thus to normalize the amounts loaded onto different gel lanes. To investigate this practical application, a complex cellular sample was used. This sample underwent extraction using one of four different methods. Two of these methods (urea and native) were compatible with a standard assay, while the other two (SDS and RIPA buffers) required the cyclodextrin-containing assay. With each method, the process was conducted twice, beginning with different cell pellets and ending by loading a theoretically identical amount of sample onto the gel. The results are shown in Fig 7. Integration of the staining profile for each line shows that the amounts loaded varied by less than 10%, which appears reasonable for a biochemical technique.

After extraction, involving two independent extractions from different cell pellets, the protein extracts were assayed using the standard Bradford assay (urea and native extracts), the Bradford assay in the presence of 2.5 mg/ml alpha-cyclodextrin (cuvette format, 10 μl of sample) for the SDS extract, and the Bradford assay in the presence of 5 mg/ml alpha-cyclodextrin (cuvette format, 10 μl of sample) for the RIPA extract.

Following the protein assays, the indicated amounts of proteins were loaded on top of the gel lanes. The proteins were separated by electrophoresis and the gel stained by Coomassie blue. The values at the bottom of each lane correspond to the integration of the lane profile using Image J software.

As a conclusion, the optimized method for protein assay in the presence of SDS or RIPA buffer is summarized in Tables 4 and 5.

**Table 4:** Flowchart for protein assay in the cuvette format.

Reagent preparation
For samples in Laemmli buffer: add 20μl of alpha-cyclodextrin solution (125 mg/ml) per ml of Bradford reagent. Mix well*
For samples in RIPA buffer: add 40μl of alpha-cyclodextrin solution (125 mg/ml) per ml of Bradford reagent. Mix well*
Sample preparation
Dilute the samples and the BSA standards to the appropriate concentration in Laemmli (or RIPA) buffer For samples, 1x 3x and 10x dilutions usually give at least one useful measurement
Assay procedure
Pipet 1ml of complete reagent (with cyclodextrin) in a microcentrifuge tube. Add 10μl of sample or BSA standard. Mix by inversion of the tube.
After 5 minutes, read the absorbance at 595 nm. Build the standard curve and use it to determine the protein concentration of the sample(s) of interest. A visual comparison of the sample-containing tubes with the standard-containing wells prior to absorbance reading is recommended. If the samples blue color is more pronounced than the color of the most concentrated BSA standard, then a further dilution of the sample is to be performed and assayed. This is made possible by the stability time range of the assay (>1 hour)

* the complete reagent is stable for at least one week if stored at +4°C

**Table 5:** Flowchart for protein assay in the microplate format. For samples in Laemmli buffer, the scale of the assay (5 or 10 μl sample) must be determined first. The only practicable scale for samples in RIPA buffer is 5 μl of sample.

Reagent preparation
For 5 μl samples in Laemmli buffer: for each ml of complete reagent, mix 40μl of alpha-cyclodextrin solution (125 mg/ml) with 960 μl of Bradford reagent. Mix well
For 5 μl samples in RIPA buffer or 10 μl samples in Laemmli buffer: for each ml of complete reagent, mix 80μl of alpha-cyclodextrin solution (125 mg/ml) with 920 μl of Bradford reagent. Mix well
Sample preparation
Dilute the samples and the BSA standards to the appropriate concentration in Laemmli (or RIPA) buffer For BSA standards, a 0.1 to 2 mg/ml range is recommended For samples, 1x 3x and 10x dilutions usually give at least one useful measurement
Assay procedure
Pipet 250 μl of complete reagent (with cyclodextrin) per well in the microplate. Depending of the assay format, add 5 μl or 10 μl of sample or BSA standard. Mix by repeated pipetting.
After 5 minutes, read the absorbance at 595 nm. Build the standard curve and use it to determine the protein concentration of the sample(s) of interest. A visual comparison of the sample-containing wells with the standard-containing wells prior to absorbance reading is recommended. If the samples blue color is more pronounced than the color of the most concentrated BSA standard, then a further dilution of the sample is to be performed and assayed. This is made possible by the stability time range of the assay (>1 hour)

Note: The Bradford reagent stains the plastic reservoirs used for multichannel plates. The reservoirs are easily cleaned with 95% ethanol followed by ultrapure water.

## Discussion

Compared to the initial version of the assay [15], the modified method reverses the concentration of the reactants. The initial version used a diluted cyclodextrin solution, requiring concentrated Bradford reagent had to be used, which was corrosive and viscous and, therefore, difficult to use. In the present version of the assay, an innocuous, low viscosity, concentrated alpha-cyclodextrin solution is used, in conjunction with any normal strength commercial or laboratory-prepared Bradford reagent. As cyclodextrins remove SDS efficiently, even from proteins [27], it was anticipated that contact of the sample with the cyclodextrin prior to addition of the Bradford reagent may result in premature SDS removal and thus protein precipitation on the side of the container used for the assay (tube or microplate well). This is why the preparation of a complete cyclodextrin-Bradford reagent has been favored. The high concentration of alpha-cyclodextrin that can be dissolved in the stock solution means that the resulting dilution of the Bradford reagent is negligible and has no consequences for the assay, even using the microplate format.

With regard to the optimal cyclodextrin type, gamma-cyclodextrin was not tested because of its ability to bind proteins [28]. Alpha-cyclodextrin was found to be much more practical to use because of its high water solubility, enabling easy use with limited reagent dilution, and low interference with the assay response curve, provided that the cyclodextrin/detergent ratio was carefully optimized, a dimension that had not been investigated in the previous version of the assay.

Practically speaking, the best protein range for the assay was 2–10 μg protein. This means a protein concentration range of 0.2–1 mg/ml in Laemmli SDS buffer for an assay with a 10-μl sample volume. This range is often reached for total extracts from biological samples. As an example, the RAW264 cell extracts used had a protein concentration of 5–10 mg/ml and required a 10-fold dilution in SDS buffer prior to the assay. As the color develops in 5 minutes and is stable for one hour, any sample that is above the upper linear limit can easily be assayed by further dilution in SDS buffer.

For samples that are below the 0.1 mg/ml protein concentration limit, a 20-μl sample volume can be tolerated if the cyclodextrin concentration in the assay is increased to 5 mg/ml. As shown in Fig 3, this increase does not alter the standard curve. Therefore, it may not be necessary to completely rerun the assay in the case of a single low concentration point, at the expense of a possible minor loss in the accuracy of the assay. It is however recommended to run the assay with a fixed amount of SDS (or RIPA) buffer, especially in the microplate format, to keep the consistency of the assay and to limit variability. Even in duplicates, the coefficient of variation of the assay is usually lower than 5%, both in the cuvette and microplate format, and exactly in the same range for the standard assay without cyclodextrins and the cyclodextrin-containing assay.
